# PFAS Exposure and Endocrine Disruption Among Women

**DOI:** 10.1001/jamanetworkopen.2025.39425

**Published:** 2025-12-05

**Authors:** Rezaul Karim Ripon, Md Jamil Hossain, Mayra Volquez, Elizabeth Meda-Monzon, Sujata Saunik, Narayana Prasad

**Affiliations:** 1 Environmental Epidemiology, Harvard T.H. Chan School of Public Health, Boston, Massachusetts; 2 McHigher Center for Health Research, Dhaka, Bangladesh; 3 Public Health Literacy, Brigham and Women’s Hospital, Harvard Medical School, Boston, Massachusetts; 4 Department of Public Health and Informatics, Jahangirnagar University, Dhaka, Bangladesh; 5 Public Health Literacy, UT Southwestern, Miami, Florida; 6 Pharmamanagement EAS De RL, Mexico City, Mexico; 7 Takemi Fellow, Harvard T.H. Chan School of Public Health, Boston, Massachusetts

## Abstract

This cross-sectional study investigates the association between perfluoroalkyl and polyfluoroalkyl substances (PFAS) exposure and endocrine disruption among US women.

## Introduction

Perfluoroalkyl and polyfluoroalkyl substances (PFAS) are commonly used in industrial and consumer products because of their heat resistance and hydrophobic and oleophobic properties.^1^ The primary sources of widespread PFAS exposure are contaminated drinking water, food, indoor dust, outdoor air, and soil.^1^ Nearly everyone in the US has detectable levels of PFAS in their blood.^1^ PFAS analytes can alter hormone secretion, menstrual cyclicity, and fertility and affect reproductive tissues directly or indirectly through endocrine disruption (ED).^2^ Despite increasing evidence associating single PFAS exposure with ED, a mixture of PFAS may hamper the association.^3^ However, there is limited evidence of the association of PFAS mixture exposure with ED. This study examines the association between single and mixture PFAS analytes exposure and ED among US women.

## Methodology

This cross-sectional study was approved by the National Center for Health Statistics Ethics Review Board and is reported in accordance with the STROBE reporting guideline. The study population was from the National Health and Nutrition Examination Survey from 2015 to 2020. The primary outcome, ED, was defined as a self-reported presence or absence of estrogen or progesterone hormone or both intake. ED was used as a proxy measure for exposure to exogenous estrogen or progesterone, not for clinically diagnosed ED. Primary exposures included serum concentration of 7 PFAS analytes (Me-PFOSA-AcOH, PFNA, PFUnDA, n-PFOA, Sb-PFOA, n-PFOS, and sm-PFOS). Covariates included self-reported race and ethnicity, smoking status, physical activity, alcohol use, blood pressure, cholesterol level, age, family poverty income ratio, and body mass index (BMI; calculated as weight in kilograms divided by height in meters squared). Before data preparation, we excluded any women with missing ED or survey design variable information We used multiple imputation techniques to adjust 20 women with missing BMI. For primary analyses of associations between individual PFAS analyte exposure and ED, we used survey-weighted logistic regression. We used restricted cubic splines for PFAS when possible, otherwise using linear terms. We then conducted Bayesian kernel machine regression (BKMR) to assess the association of multiple PFAS exposures with ED. Further sensitivity analyses were undertaken to test robustness (eMethods in Supplement 1). Statistical analyses were performed using R statistical software version 4.3.0 (R Project for Statistical Computing). Statistical significance was set at a 2-sided *P* < .05.

## Results

Among 1995 women (median [IQR] age, 56 [31-74] years; 12.5% Black, 8.5% Mexican American, 6.2% other Hispanic, and 63.7% White), 15.8% individuals with ED were identified. Participants with ED were more likely to be non-Hispanic White and older and had higher median concentrations of several PFAS compounds (M Me-PFOSA-AcOH, PFNA, PFOA, PFOS, and sm-PFOS) (Table 1). In regression spline models, n-PFOS showed the highest adjusted estimate in the association with ED, with 10.8 (95% CI, 6.41-15.2) at the first spline, while other PFAS (PFNA and Me-PFOSA-AcOH) also had positive associations with smaller estimates across several spline terms. In BKMR, increasing all PFAS mixture exposures from the 25th to 75th percentile changed associated odds ratios of ED from 0.82 (95% CI, 0.78-0.84) to 1.49 (95% CI, 1.36-1.67); sm-PFOS had the highest posterior inclusion probability (0.95) (Table 2).

**Table 1.** Survey-Weighted Descriptive Analysis of Study Population

Variable	Women, % (95% CI)			*P* value
	Total	No ED	ED	
Activity level				
Highly active	29.2 (26.5-31.9)	23.2 (15.1-31.3)	30.6 (27.4-33.7)	.09
Inactive	38.8 (36.0-41.6)	47.2 (39.5-54.9)	36.9 (33.6-40.2)	
Moderately active	32.0 (28.8-35.2)	29.6 (22.1-37.1)	32.5 (29.4-35.7)	
Age, median (IQR), y	56 (31-74)	45 (59-31)	67 (74-55)	<.001
Alcohol use				
Current drinker	65.7 (58.7-72.6)	68.3 (54.4-82.1)	65.1 (57.5-72.7)	.26
Former drinker	17.1 (12.9-21.4)	20.9 (9.6-32.2)	16.2 (11.8-20.7)	
Never drinker	17.2 (12.9-21.5)	10.9 (5.8-16.0)	18.7 (13.7-23.7)	
BMI, median (IQR)	28.35 (23.9-33.9)	28.10 (33.90-23.90)	28.60 (33.40-24.10)	.94
Race and ethnicity^a^				
Mexican American	8.5 (5.9-11.1)	3.3 (1.7-5.0)	9.7 (6.7-12.7)	<.001
Non-Hispanic Black	6.2 (4.4-8.0)	3.5 (1.7-5.4)	6.8 (4.7-8.9)	
Non-Hispanic White	63.7 (58.8-68.7)	80.7 (75.7-85.8)	59.8 (54.2-65.4)	
Other Hispanic	12.5 (9.2-15.8)	6.6 (4.1-9.1)	13.9 (10.3-17.4)	
Other, including multiracial or multiethnic	9.1 (7.1-11.1)	5.8 (2.2-9.5)	9.8 (7.7-12.0)	
Smoking status				
Yes	36.6 (33.3-39.9)	41.5 (33.1-49.8)	35.5 (32.3-38.7)	.20
No	63.4 (60.1-66.7)	58.5 (50.2-66.9)	64.5 (61.3-67.7)	
PFAS exposure level, median (IQR), ng/mL				
Me-PFOSA-AcOH, ng/mL	0.09 (0.07-0.20)	0.07 (0.20-0.07)	0.10 (0.20-0.07)	.03
PFNA, ng/mL	0.55 (0.30-1.10)	0.50 (0.80-0.30)	0.60 (1.10-0.40)	.01
PFOA, ng/mL	1.50 (0.70-2.70)	1.20 (1.90-0.70)	1.80 (2.70-1.20)	.004
PFOA isomer, ng/mL	0.07 (0.07-0.08)	0.07 (0.07-0.07)	0.07 (0.07-0.08)	.24
PFOS, ng/mL	3.05 (1.30-7.00)	2.30 (3.80-1.30)	3.80 (7.00-2.30)	<.001
PFUnDA, ng/mL	0.09 (0.07-0.20)	0.07 (0.20-0.07)	0.10 (0.20-0.07)	.82
Sm-PFOS, ng/mL	1.35 (0.50-3.20)	0.90 (1.70-0.50)	1.80 (3.20-1.00)	<.001

Abbreviations: BMI, body mass index (calculated as weight in kilograms divided by height in meters squared); ED, endocrine disruption; Me-PFOSA-AcOH, *N*-methyl-perfluorooctane sulfonamido acetic acid; PFAS, perfluoroalkyl and polyfluoroalkyl substances; PFNA, perfluorononanoic acid; PFOA, perfluorooctanoic acid; PFOS, perfluorooctane sulfonic acid; PFUnDA, perfluoroundecanoic acid; sm-PFOS, sum of perfluoromethylheptane sulfonic acid isomers.

^a^ Race and Hispanic origin categories were Mexican American, non-Hispanic Black, non-Hispanic White, other Hispanic, and other race and ethnicity, including multiracial and multiethnic. Race and ethnicity were assessed to describe the study population and to adjust for potential confounding in the analysis.

**Table 2.** Association Between Single and PFAS Mixture Exposure and ED

PFAS	ED, estimate (95% CI)	
	Unadjusted	Adjusted^a^
Me-PFOSA-AcOH		
Spline 1	0.88 (–1.24 to 2.99)	–0.14 (–3.55 to 3.27)
Spline 2	2.64 (1.54 to 3.74)	2.77 (1.22 to 4.32)
Spline 3	2.73 (0.94 to 4.52)	3.17 (0.11 to 6.23)
PFNA		
Spline 1	2.19 (0.88 to 3.50)	2.95 (0.78 to 5.11)
Spline 2	3.32 (1.78 to 4.86)	3.77 (1.41 to 6.13)
Spline 3	3.37 (0.99 to 5.75)	3.22 (0.26 to 6.18)
PFUnDA		
Spline 1	–0.55 (–4.41 to 3.31)	–0.29 (–7.35 to 6.77)
Spline 2	–0.44 (–3.07 to 2.19)	0.16 (–2.69 to 3.02)
Spline 3	–1.32 (–7.74 to 5.10)	–2.18 (–10.54 to 6.18)
n-PFOA		
Spline 1	5.19 (2.78 to 7.60)	4.67 (0.73 to 8.61)
Spline 2	3.13 (–5.96 to 12.2)	2.60 (–8.23 to 13.4)
Spline 3	–3.91 (–22.0 to 14.2)	–2.79 (–24.6 to 19.0)
n-PFOS		
Spline 1	6.13 (3.72 to 8.55)	10.8 (6.41 to 15.2)
Spline 2	–2.87 (–15.2 to 9.47)	–16.4 (–18.4 to 2.55)
Spline 3	–4.6 (–8.9 to 9.75)	–4.5 (–8.5 to –3.48)
Sm-PFOS		
Spline 1	4.40 (3.21 to 5.59)	4.62 (3.16 to 6.08)
Spline 2	3.93 (1.34 to 6.52)	2.26 (–0.86 to 5.38)
Spline 3	0.52 (–2.88 to 3.92)	–0.11 (–3.95 to 3.75)
Mixture quantile, OR^b^		
0.25 (sm-PFOS)	0.82 (0.78 to 0.84)	NA
0.75 (n-PFOS)	1.49 (1.36 to 1.67)	NA
Difference (75th to 25th)	1.82	NA

Abbreviations: ED, endocrine disruption; Me-PFOSA-AcOH, N-methyl-perfluorooctane sulfonamido acetic acid; NA, not applicable; n-PFOA, linear isomer of PFOA; n-PFOS, linear isomer of PFOS; OR, odds ratio; PFAS, perfluoroalkyl and polyfluoroalkyl substances; PFNA, perfluorononanoic acid; PFOA, perfluorooctanoic acid; PFOS, perfluorooctane sulfonic acid; PFUnDA, perfluoroundecanoic acid; sm-PFOS, sum of perfluoromethylheptane sulfonic acid isomers.

^a^ Adjusted for age, race and ethnicity, smoking status, physical activity category, alcohol use, and body mass index (calculated as weight in kilograms divided by height in meters squared).

^b^ The top 3 posterior inclusion probabilities were 0.95 for sm-PFOS, 0.64 for n-PFOS, and 0.43 for n-PFOA.

## Discussion

Data from this cross-sectional study show that exposure to single and mixtures of PFAS was associated with higher odds of ED among women. Our findings demonstrated that certain PFAS compounds, particularly n-PFOS, were associated with ED. PFAS are widely used in industry, and increasing evidence suggests that even low-level, chronic exposure may disrupt endocrine function and harm health.^1^ Exposure to mixtures of PFAS remained positively associated with developing ED. One study found that exposure to a combination of PFAS was associated with disrupted lipid and amino acid metabolism and altered thyroid hormone function.^2,4^ PFAS mimic fatty acids and interfere with hormone-binding proteins, particularly in thyroid hormone transport,^5^ which may explain the association of PFAS mixtures with ED. In this association, sm-PFOS was the highest contributing factor. Study limitations include the cross-sectional design, which means we cannot rule out reverse causation, whereby ED status may influence PFAS concentrations through altered pharmacokinetics. Additionally, defining ED as exogenous hormones could reflect indications other than ED (menopausal symptoms or prevention of osteoporosis), which may contribute to misclassification. In addition, n-PFOS appeared protective against ED likely due to confounding factors, random variation, or model limitations rather than a true biological association.

## Notes

Supplement 1.
eMethods.

Supplement 2.
Data Sharing Statement
