# Supplementary material for: PFAS Exposure and Endocrine Disruption Among Women
Source: JAMA Netw Open. 2025 Dec 5;8(12):e2539425. doi: 10.1001/jamanetworkopen.2025.39425 (PMC12681035; doi:10.1001/jamanetworkopen.2025.39425)
Supplement: Supplement 1. — eMethods. [file jamanetwopen-e2539425-s001.pdf]

Supplemental Online Content

Ripon RK, Hossain MJ, Volquez M, Meda-Monzon E, Saunik S, Prasad N. PFAS exposure and endocrine disruption among women. *JAMA Netw Open*. 2025;8(10):e2539425. doi:10.1001/jamanetworkopen.2025.39425

eMethods.

This supplemental material has been provided by the authors to give readers additional information about their work.

© 2025 Ripon RK et al. *JAMA Network Open*.

eMethods.

We calculated standardized descriptive statistics, incorporating survey weights. A bivariate analysis is conducted using survey-weighted t-tests and Rao-Scott chi-square tests. We had only 20 missing data on BMI. Missing data on BMI were addressed through multiple imputation by chained equations using predictive mean matching. A multiple (m=5) imputed dataset was generated, and the first imputed dataset was used in the analysis.

Outcome Assessment: The primary outcome for this analysis was ED. This outcome self-reported use of female hormones, as assessed by questionnaire item RHQ540 in the NHANES Reproductive Health component. Participants were classified as 'ED' if they answered "yes" to the question, "Have you ever used female hormones such as estrogen and progesterone? Please include any forms of female hormones, such as pills, cream, patch, and injectables, but do not include birth control methods or use for infertility." Participants who indicated use of female hormones exclusively for birth control or infertility were not included as hormone users.

Exposure Assessments: The primary exposures of interest were serum concentrations of PFAS analytes, measured in participants' blood samples as part of the NHANES laboratory component. PFAS included 2-(N-methyl-PFOSA) acetic acid (Me-PFOSA-AcOH), perfluorononanoic acid (PFNA), perfluoroundecanoic acid (PFUnDA), n-perfluorooctanoic acid (n-PFOA), branched perfluorooctanoic acid isomers (Sb-PFOA), n-perfluorooctane sulfonic acid (n-PFOS), and perfluoromethylheptane sulfonic acid isomers (Sm-PFOS). Laboratory analyses were conducted using online solid-phase extraction coupled to high-performance liquid chromatography–tandem mass spectrometry. Values below the lower limit of detection (LOD), NHANES assigned an imputed value equal to the LOD divided by the square root of 2 (LOD/√2).

Covariates: Covariates included self-reported race/ethnicity (non-Hispanic White, non-Hispanic Black, Hispanic, and other), cigarette smoking status (current, former, or never smoker), physical activity level(inactive, moderately active, highly active) based on total metabolic equivalent minutes per week, and alcohol use (never, former, current drinker). Age (in years) and body mass index (BMI; kg/m^2) were also included as covariates.

Statistical Model: To evaluate associations between individual PFAS serum concentrations and ED, we conducted regression analyses that accounted for the complex NHANES survey design. Serum PFAS concentrations were modeled as continuous predictors—using natural cubic spline terms to flexibly capture potential non-linear relationships—unless model convergence

© 2025 Ripon RK et al. *JAMA Network Open*.

required simpler, lower-degree splines or linear terms. ED was modeled as a binary variable using survey-weighted generalized linear models with a quasibinomial link to account for potential overdispersion. Models were estimated unadjusted and adjusted for a priori selected covariates, including age, race/ethnicity, body mass index, smoking status, physical activity level, and alcohol use. Predicted probabilities and 95% confidence intervals were generated across the observed PFAS distributions for both unadjusted and adjusted models. We also employed BKMR to evaluate the joint effect of multiple PFAS analytes on ED. BKMR flexibly models exposure–response relationships, accommodating potential non-linear and non-additive interactions among exposures. In our analysis, the exposure matrix included the PFAS of interest. At the same time, covariates (age, race/ethnicity, smoking status, physical activity, and alcohol use) were incorporated as adjustment variables. Within the BKMR framework, we modeled the probability of ED using a probit link function appropriate for binary outcomes. Variable selection was performed to estimate the PIP for each PFAS, representing the probability that a given exposure was included in the model during Markov chain Monte Carlo sampling (50,000 iterations). A PIP threshold 0.5 was used to indicate PFAS most strongly associated with the outcome. NHANES sample weights were not applied to the BKMR model, as all variables used for sample weighting were included as covariates in the adjusted model, per recommended analytic guidance. To evaluate the robustness of our findings, we performed several sensitivity analyses. These included assessing the impact of imputing missing BMI values, comparing models with alternative covariate adjustment sets, contrasting linear versus spline modeling approaches for PFAS exposures, and repeating analyses after excluding influential outliers or participants with extreme exposure values. We also compared results from survey-weighted and unweighted modes. All modeling was conducted in R (version 4.4.3).

© 2025 Ripon RK et al. *JAMA Network Open*.
