# Supplementary material for: PFAS Exposure and Endocrine Disruption Among Women
Source: JAMA Netw Open. 2025 Dec 5;8(12):e2539425. doi: 10.1001/jamanetworkopen.2025.39425 (PMC12681035; doi:10.1001/jamanetworkopen.2025.39425)
Supplement: Supplement 2. — Data Sharing Statement [file jamanetwopen-e2539425-s002.pdf]

Data Sharing Statement

Ripon. PFAS Exposure and Endocrine Disruption Among Women. *JAMA Netw Open*. Published online October 27, 2025 doi:10.1001/jamanetworkopen.2025.39425

Data
Data available: Yes
Data types: Data dictionary
How to access data: The data are available here: https://wwwn.cdc.gov/nchs/nhanes/**When available:** With publication

Supporting Documents
Document types: None

Additional Information
Who can access the data: Upon Request
Types of analyses: Upon Request
Mechanisms of data availability: Upon Request
